# SARS-CoV-2 Infection and Active, Multiorgan, Severe cGVHD After HSCT for Adolescent ALL: More Luck Than Understanding? A Case Report

**DOI:** 10.3389/fped.2021.775318

**Published:** 2022-01-14

**Authors:** Natalia Zubarovskaya, Irene Hofer-Popow, Marco Idzko, Oskar A. Haas, Anita Lawitschka

**Affiliations:** ^1^Stem Cell Transplant Unit, St. Anna Children's Hospital, Medical University of Vienna, Vienna, Austria; ^2^Department of Pulmonology, Medical University of Vienna, Vienna, Austria; ^3^Labdia Labordiagnostik GmbH, Vienna, Austria; ^4^St. Anna Children's Cancer Research Institute, Vienna, Austria; ^5^St. Anna Children's Hospital, Pediatric Clinic, Medical University of Vienna, Vienna, Austria

**Keywords:** COVID-19, stem cell transplantation, immunodeficiency, chronic graft vs. host disease, SARS-CoV-2

## Abstract

Graft-vs. -host disease (GvHD) is a serious and complex immunological complication of haematopoietic stem cell transplantation (HSCT) and is associated with prolonged immunodeficiency and non-relapse mortality. Standard treatment of chronic GvHD comprises steroids in combination with other immunosuppressive agents. Extracorporeal photopheresis (ECP), with its immunomodulatory mechanism, is applied as part of steroid-sparing regimens for chronic GvHD. Immunocompromised, chronically ill patients are at particular risk of severe disease courses of severe acute respiratory syndrome coronavirus 2 (SARS-CoV-2) infection. T-cell immunity in SARS-CoV-2 infection is well-described but the role of the humoral immune responses is not fully understood. This case report describes a moderate course of SARS-CoV-2 infection in a patient <9 months after HSCT who was suffering from active, severe, chronic GvHD treated with prednisone and ECP. Following HSCT from a matched unrelated donor to cure acute lymphoblastic leukaemia, the 25-year-old male patient experienced multiple infectious complications associated with cytopenia, B-cell dyshomeostasis and autoantibody production followed by development of severe chronic GvHD thereafter at day +212. The steroid-sparing treatment plan consisted of supportive care, topical treatment, prednisone and ECP. He was diagnosed with SARS-CoV-2 infection at day +252, experiencing loss of smell and taste as well as a cough. The patient's oxygen saturation was between 94 and 97% on room air, and computed tomography images showed evolution of typical of SARS-CoV-2 infiltrates. In addition to cytopenia and immune dyshomeostasis, laboratory tests confirmed macrophage activating syndrome, transaminitis and Epstein-Barr virus viraemia. At that time, anti-SARS-CoV-2 monoclonal antibodies were not available in Austria and remdesivir seemed contraindicated. Surprisingly, despite severe lymphopenia the patient developed SARS-CoV-2-specific antibodies within 15 days, which was followed by clearance of SARS-CoV-2 and EBV with resolution of symptoms. Thereafter, parameters of immune dysregulation such as lymphopenia and B-cell dyshomeostasis, the latter characterised by elevated CD21^low^ B cells and autoantibody expression, normalised. Moreover, we observed complete response of active chronic GvHD to treatment.

## Introduction

Chronic graft-vs.-host disease (cGvHD) is the most common complication after allogeneic haematopoietic stem cell transplantation (HSCT), affecting 30–70% of successfully transplanted patients (1, 2). The treatment of this complex condition, which resembles various autoimmune diseases, remains challenging due to various clinical phenotypes and the multiplicity of organ-specific medical complications. Treatment relies on prolonged immunosuppression, which—in addition to the immunodeficient effects of the disease itself—increases the risk of infection resulting in high morbidity and mortality. Immunocompromised patients are at particular risk of developing severe viral infection caused by severe acute respiratory syndrome coronavirus 2 (SARS-CoV-2), also known as coronavirus disease 2019 (COVID-19) (3).

The preventive measures and treatment of transplanted patients with SARS-CoV-2 are based on The European Society for Bone and Marrow Transplantation (EBMT) guidelines (4).

Severe acute respiratory syndrome caused by SARS-CoV-2 infection is responsible for the 28% mortality rate associated with this infection in older patients and 9% mortality rate seen in children after HSCT (5). In this context, T-cell immunity is the main focus of recently published studies (6), while the role of adaptive immune responses has been described to a lesser extent. In the last 20 years, B-cell dyshomeostasis and an accumulation of several circulating CD21^low^ B-cell populations has been described in different disease entities associated with chronic immune stimulation (such as viral or parasite infection), in common variable immunodeficiencies with immune dysregulation, in GvHD and in autoimmune diseases (7–10). Recently, Oliviero et al. reported on the expansion of atypical memory B cells (CD21^low^/CD27^−^/CD10^−^) in non-immunocompromised patients with SARS-CoV-2 infection in terms of both frequency and cell number in contrast to healthy donors and re-convalescents (11). Additionally, a negative correlation between the proportion of atypical memory B cells and survival was found.

Various transplant centres and transplant societies have published their experience with SARS-CoV-2 infection in the HSCT setting (12). Here we report a case of SARS-CoV-2 infection 9 months after HSCT using a matched unrelated donor (MUD) in a male patient with acute lymphoblastic leukaemia (ALL) suffering from active cGVHD with serious immune dyshomeostasis and systemic immunosuppression.

## Materials and Methods

The patient's medical report included clinical features, laboratory tests, radiographic imaging, treatment schedules and description of outcome. The detection of SARS-CoV-2 RNA in a nasopharyngeal swab and Epstein–Barr virus (EBV) DNA in peripheral blood was based on real-time polymerase chain reaction (PCR), which is routinely used in our clinic. Neutralising SARS-CoV-2 antibodies were measured by the Elecsys Anti-SARS-CoV-2 S immunoassay (Roche Diagnostics). The thresholds used were <0.80 binding antibody units (BAU) for negativity and >15 BAU neutralising IgG antibodies for positivity (13). Flow cytometry was used for assessment of numbers of B and T lymphocytes, monocytes and natural killer (NK) cells.

Engraftment after HSCT was defined as the time point of a sustained peripheral blood neutrophil count of >500 × 10^6^/L on 3 consecutive days (14) and independency from transfusion for at least 7 days with a platelet count of more than >20,000 × 10^9^/L and a haemoglobin level of ≥7 g/dL (15).

We categorised the severity of SARS-CoV-2 infection using recent guidance from the US National Institutes for Health (NIH) (16). The disease course of SARS-CoV-2 infection can be divided into asymptomatic, mild, moderate, severe and critical presentations. A mild course is characterised by symptoms such as fever, cough, sore throat, malaise, headache, muscle pain, nausea, vomiting, diarrhoea, loss of taste and smell without dyspnoea, and abnormal chest imaging; a moderate course is characterised by lower respiratory tract involvement assessed by clinical and imaging examination, with oxygen saturation (SpO_2_) ≥94% on room air; a severe course is characterised by SpO_2_ <94% on room air, a ratio of arterial partial pressure of oxygen to fraction of inspired oxygen (PaO_2_/FiO_2_) <300 mmHg, respiratory frequency >30 breaths/min, or lung infiltrates in more than 50% of lung parenchyma. A critical course presents with respiratory failure, septic shock and/or multiple organ dysfunction or failure (16).

## Case Report

Herein, we describe the clinical course of SARS-CoV-2 infection in a 25-year-old male patient of Turkish origin with ALL treated with one course of rituximab and several courses of blinatumomab followed by HSCT with peripheral blood stem cells from an MUD. The myeloablative conditioning regimen comprised 12 Gray total body irradiation and a single dose of etoposide 60 mg/kg. For prophylaxis against GvHD, anti-thymocyte globulin (ATG), cyclosporine A and methotrexate were applied.

HSCT-associated toxicity was unremarkable with mucositis [World Health Organization (WHO) grade III], febrile neutropenia, mild skin toxicity (WHO grade I) and human herpes virus 6 (HHV6) - and herpes simplex virus 1 (HSV1)-triggered mucositis. The patient developed acute skin GvHD of grade II that responded with complete resolution to treatment with a short course of prednisone 2 mg/kg/day and topical therapy with methylprednisolone and pimecrolimus. Cyclosporine A was discontinued at day +67 because of resolution of acute GvHD, prolonged engraftment kinetics and recurrent HHV6 infection. Bone marrow aspiration at day +100 showed complete remission with 100% donor chimerism in all cell populations and sufficient engraftment with the patient being transfusion independent from day +70 onwards.

The subsequent post-transplant phase was complicated by recurrent infections such as HSV1, HHV6/7 mucositis (WHO grade III) and sinusitis (without pathogen detection) accompanied by phases of pancytopenia. Additionally, the patient experienced moderate toxic nephropathy, fatigue syndrome, nausea and malnutrition; he was admitted to hospital at day +130. The microbiological evaluation of stool showed colonisation with *Klebsiella pneumoniae* that was extended-spectrum β-lactamase (ESBL)-producing and resistant to three antibiotic groups (3MRGN). A routine platelet transfusion given in the context of gastrointestinal endoscopy for exclusion of GvHD was complicated by an immunoglobulin (Ig) E-mediated reaction against plasma proteins. Platelet antibody tests were negative during the whole disease course. Nutritional support with a nasogastric tube, combined antimicrobial and antiviral therapy, and stimulation with granulocyte colony-stimulating factor (G-CSF) were started. The infectious complications and pancytopenia improved slowly but severe thrombocytopenia (thrombocytes 20 × 10^9^/L) persisted without the development of any haemorrhagic syndrome.

At day +150 immune dyshomeostasis was diagnosed, characterised by low levels of circulating CD19^+^ B cells (21 × 10^6^/L), elevated levels of IgM, an elevated percentage of CD 21^low^ B cells (30%), and the expression of multiple autoantibodies such as thyroid-, cardiolipin-, β-2 glycoprotein- and glutamic acid decarboxylase autoantibodies. Impaired T-cell reconstitution presented as low levels of CD3^+^ T cells (197 × 10^6^/L), CD3^+^CD4^+^ T cells (50 × 10^6^/L), CD3^+^CD8^+^ T cells (103 × 10^6^/L) and inversion of CD8/CD4 ratio; CD56^+^CD16^+^CD3^−^ natural killer (NK) cells were in the normal range; ferritin was elevated to 5.111 μg/dL (Table 1). Minimal residual disease was not detected. Based on the clinical course, with a Karnofsky performance score of 70%, and laboratory results, graft dysfunction with signs of immune dysregulation and inflammation triggered by recurrent infectious complications was suspected. As part of an individualised treatment plan that took into account the high risk of infection and relapse and immune dysfunction with autoantibody development (possibly followed by GvHD), we aimed for a steroid-sparing regimen: one-time plasma exchange, high-dose intravenous immunoglobulin (IVIg) substitution with steroid pre-medication (5 mg/kg) and extracorporeal photopheresis (ECP) twice weekly. All clinical symptoms improved and the patient was discharged at day +172.

**Table 1:** Dynamic selected laboratory parameters during follow up after HSCT.

	**Diagnosis of B-cell dyshomeostasis**	**Infection-triggered impairment**		**Diagnosis of cGvHD**	**Diagnosis of SARS-CoV-2**	**Recovery from SARS-CoV-2 and B-cell dyshomeostasis**
**Parameters**	**day +100**	**day +150**	**day +180**	**day +212**	**day +252**	**day +275**
CD3^+^ T cells × 10^6^/L	196	197	223	282	92	247
CD3^+^CD4^+^ T cells × 10^6^/L	41	50	58	100	26	94
CD3^+^ CD8^+^ T cells × 10^6^/L	112	103	130	154	58	128
CD19^+^ B cells × 10^6^/L	6.3	21	36	65	46	131
IgD^+^CD27^+^ B cells, %	–	5.2	–	4.8	–	5.2
IgD^−^CD27^+^ B cells, %	–	17	–	6.8	–	6.5
CD21^low^ B cells, %	–	**30**	–	**23**	–	**14**
CD56^+^CD16^+^CD3^−^NK cells x 10^6^/L	–	213	183	169	55	89
Leukocytes, g/L	3,500	2,140	1,540	1,410	2,940	3,560
ANC, g/L	1,950	1,530	1,160	1,760	2,940	2,650
Thrombocytes, g/L	169	19	22	62	24	46
Aspartate transaminase, U/L	76	118	121	148	200	86
Alanine aminotransferase, U/L	137	247	280	357	772	346
Gamma glutamyl transferase U/L	152	402	360	1,153	2,536	1,929
C-reactive protein, mg/dL	1.2	2.3	0.8	1.1	1,4	1,1
Ferritin, μg/dL	–	5.111	4.151	5.357	17.839	6.771
IgG, mg/dL	–	977	384	783	542	480
IgA, mg/dL	–	24	13	22	15	16
IgM, mg/dL	–	241	51	111	103	170
Anti-cardiolipin Ab	Pos.	Pos.		Neg.		Neg.
Anti-beta-2-glycoprotein Ab	Pos.	Pos.		Neg.		Neg.
Anti-glutamic acid decarboxylase Ab	Neg.	Pos.		Neg.		Neg.
Antinuclear Ab	Neg.	Neg.		Pos.		Neg.

*ANC, absolute neutrophil count; cGvHD, chronic graft-vs.-host disease; HSCT, haematopoietic stem cell transplantation; Ig, immunoglobulin; NK, natural killer*.

**Figure 1:**
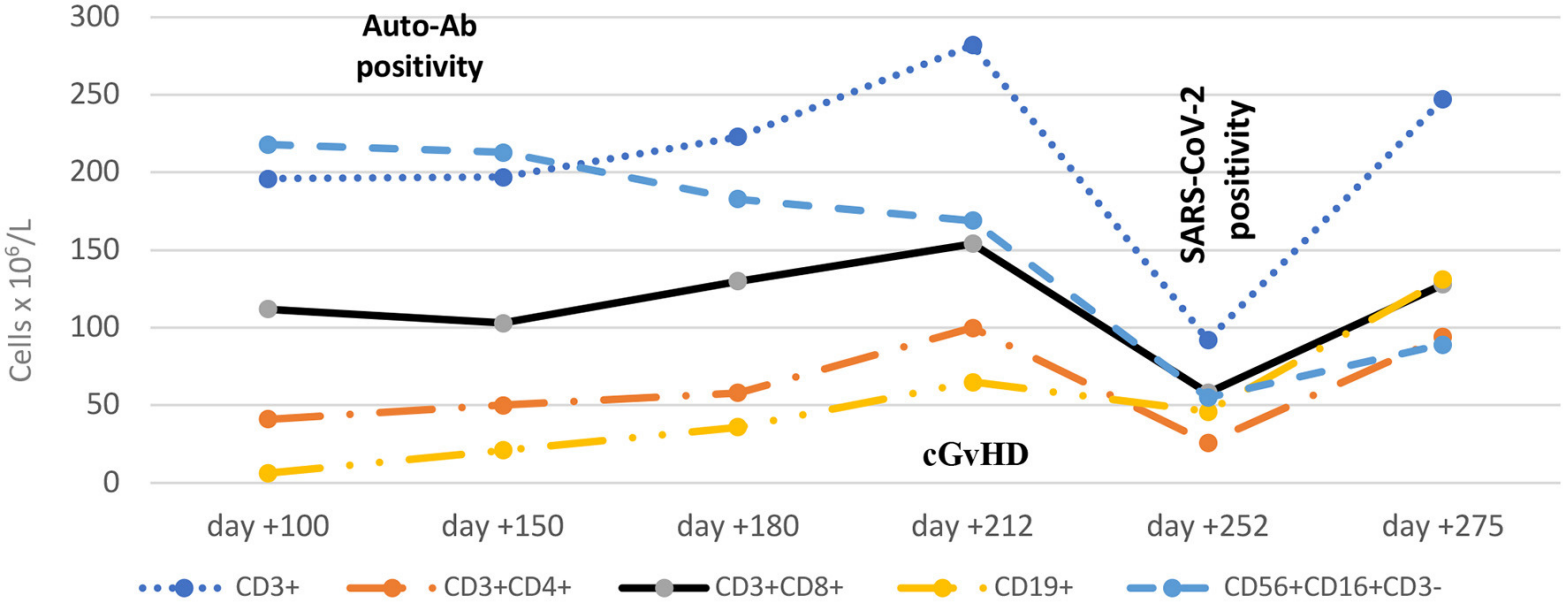
Dynamic engraftment parameters during follow up after HSCT. The time of first auto-Ab detection (day +100), diagnosis of cGvHD (day +212) and diagnosis of SARS-CoV-2 infection (day +252) are shown. Ab, antibody; cGvHD, chronic graft-vs.-host disease.

A few weeks later, at day +212 and most probably triggered by the infectious complications, the patient developed overall severe NIH-defined cGvHD with the following organ-specific scoring: fasciitis score 2 with painful periarthritis, skin score 1 and eye score 2. Liver involvement presented as transaminitis partly interpreted as a steroid-induced side effect. Parameters of the B-cell compartment revealed ongoing CD19^+^ B-cell deficiency (65 × 10^6^/L), low CD27^+^ memory B cells associated with diminished non-class- and class-switched memory B-cell subsets (IgD^+^CD27^+^ and IgD^−^CD27^+^ B cells) and elevated circulating CD21^low^ B cells. Parameters of the T-cell compartment showed similarly low levels to those observed on day +150 (Figure 1; Table 1).

The patient's personalised cGvHD management plan comprised prednisone (starting at 2 mg/kg/day then tapered to 1 mg/kg/day), IVIg substitution with pre-medication, and topical treatment with steroids, pimecrolimus and moisturisation. Supportive care included trimethoprim 160 mg/day split over two doses, three times a week; ambisome 5 mg twice a week and valganciclovir 900 mg/day split over two doses. IVIg substitution with pre-medication had to be discontinued due to an immediate adverse reaction.

Surprisingly, the patient's cGvHD responded within 4 weeks with a very good partial response in terms of fasciitis with periarthritis and skin manifestations, and complete response in terms of ocular cGvHD. The patient's status improved considerably, with a Karnofsky score of 80–90% followed by increasing psychosocial re-integration.

During the third pandemic wave in Austria in March 2021, the patient was identified at day +252 as being SARS-CoV-2 positive by real-time PCR together with his unvaccinated family. Due to his complex post-transplant course and active cGvHD, the patient was unvaccinated also. A nasopharyngeal swab revealed the variant B1.1.7 of SARS-CoV-2 with 2 × 10^9^ copies/mL at diagnosis. Clinical symptoms were mild with a cough and loss of taste and smell but absence of fever, pulmonary and gastrointestinal symptoms. Oxygen saturation (SpO_2_) was in the range 94–97% in room air. A thoracic computed tomography scan revealed small COVID-19-typical infiltrates in both upper lung lobes. Additionally, asymptomatic Epstein-Barr virus (EBV) DNAemia (1 × 10^3^ copies/mL at maximum) was detected at the same time. Laboratory assessments at diagnosis of SARS-CoV-2 infection showed thrombocytopenia (24 × 10^9^/L), slightly decreased white blood cell count (2.9 × 10^9^/L), and profound lymphopenia: 46 × 10^6^/L CD19^+^ B cells, 92 × 10^6^ /L CD3^+^ T cells, 26 × 10^6^ /L CD3^+^CD4^+^ T cells, 58 × 10^6^ /L CD3^+^CD8^+^ T cells, an inverse CD4/CD8 ratio and low level of CD56^+^CD16^+^CD3^−^ NK cells (55 × 10^6^ /L). C-reactive protein and fibrinogen were slightly elevated and ferritin was raised to 17.839 μg/L, while procalcitonin and interleukin (IL)-6 were normal. Hyperferritinaemia and slightly increased triglycerides and increasing transaminitis led to the diagnosis of macrophage activating syndrome (MAS). Laboratory results are presented in Table 1.

In view of the patient's rising SARS-CoV-2 load and poor immune reconstitution, he was admitted to the SARS-CoV-2-specific intermediate care unit of the University Hospital Vienna. Unfortunately, the patient suffered from an anxiety syndrome aggravated by his SARS-CoV-2 infection and EBV reactivation and left the hospital within hours. Relying on his good clinical condition, the patient refused readmission to the SARS-CoV-2-specific intermediate care unit, being monitored 3 times a week by our HSCT outpatient clinic in a close, coordinated relationship with our external care service.

Therapeutic interventions were discussed within a multidisciplinary team and in accordance with the EBMT recommendations in place at that time: supportive therapy comprised antiviral prophylaxis with valganciclovir and non-steroidal anti-inflammatory drugs; treatment with remdesivir seemed contraindicated due to severe hepatopathy; in consideration of the patient's history of an IgE-mediated plasma protein-specific reaction, we refrained from treatment with re-convalescent plasma transfusions. Prednisone was reduced to 0.8 mg/kg/day in the light of impending EBV-associated lymphoproliferation. At that time, anti-SARS-CoV-2 monoclonal antibodies (mAb) were not available in our country. ECP had to be postponed for nearly 4 weeks due to logistical reasons.

**Figure 2:**
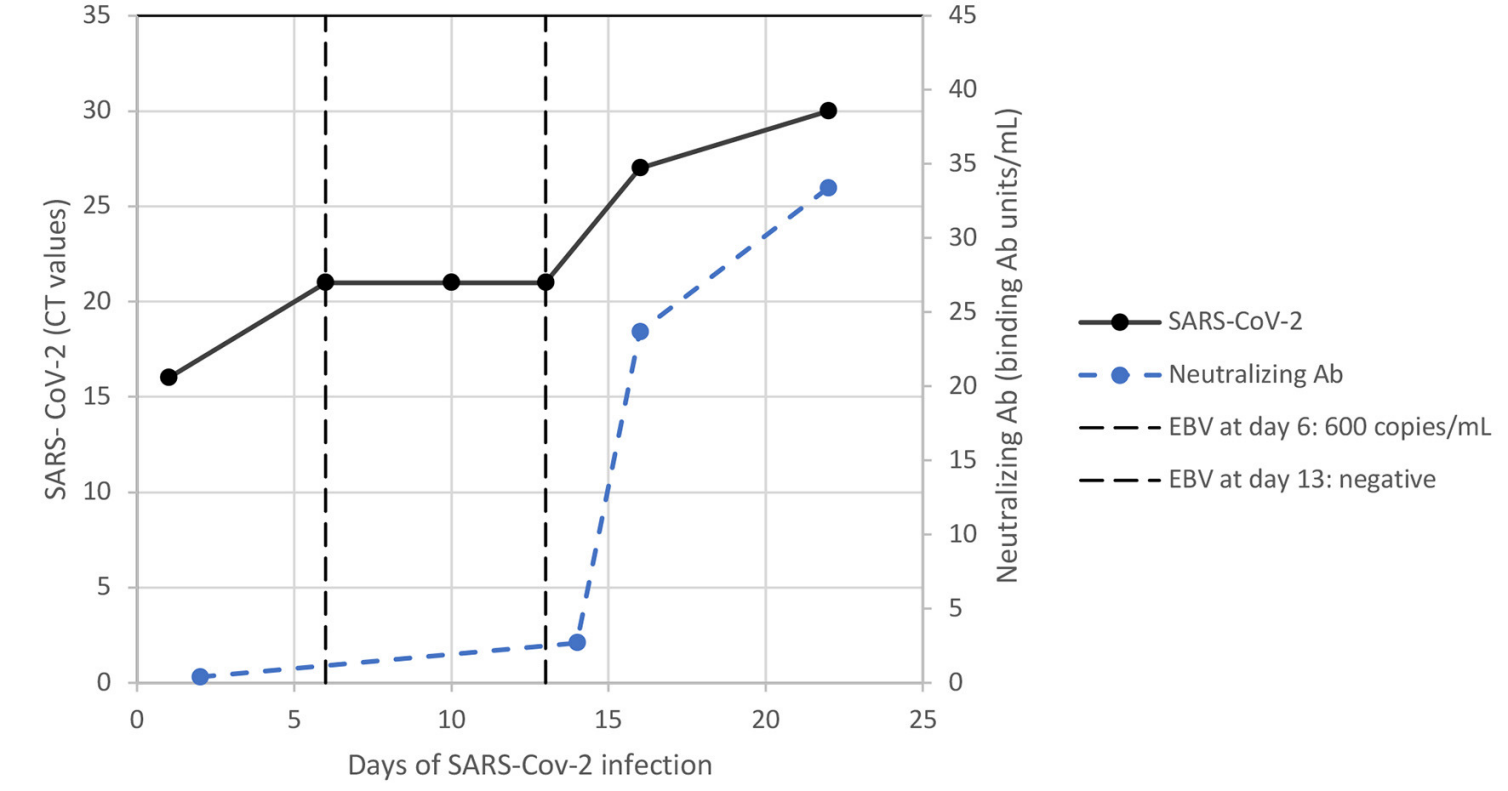
Dynamic infectious parameters of SARS-CoV-2 infection and EBV DNAemia. Ab, antibody; CT, cycle threshold; EBV, Epstein-Barr virus.

**Figure 3:**
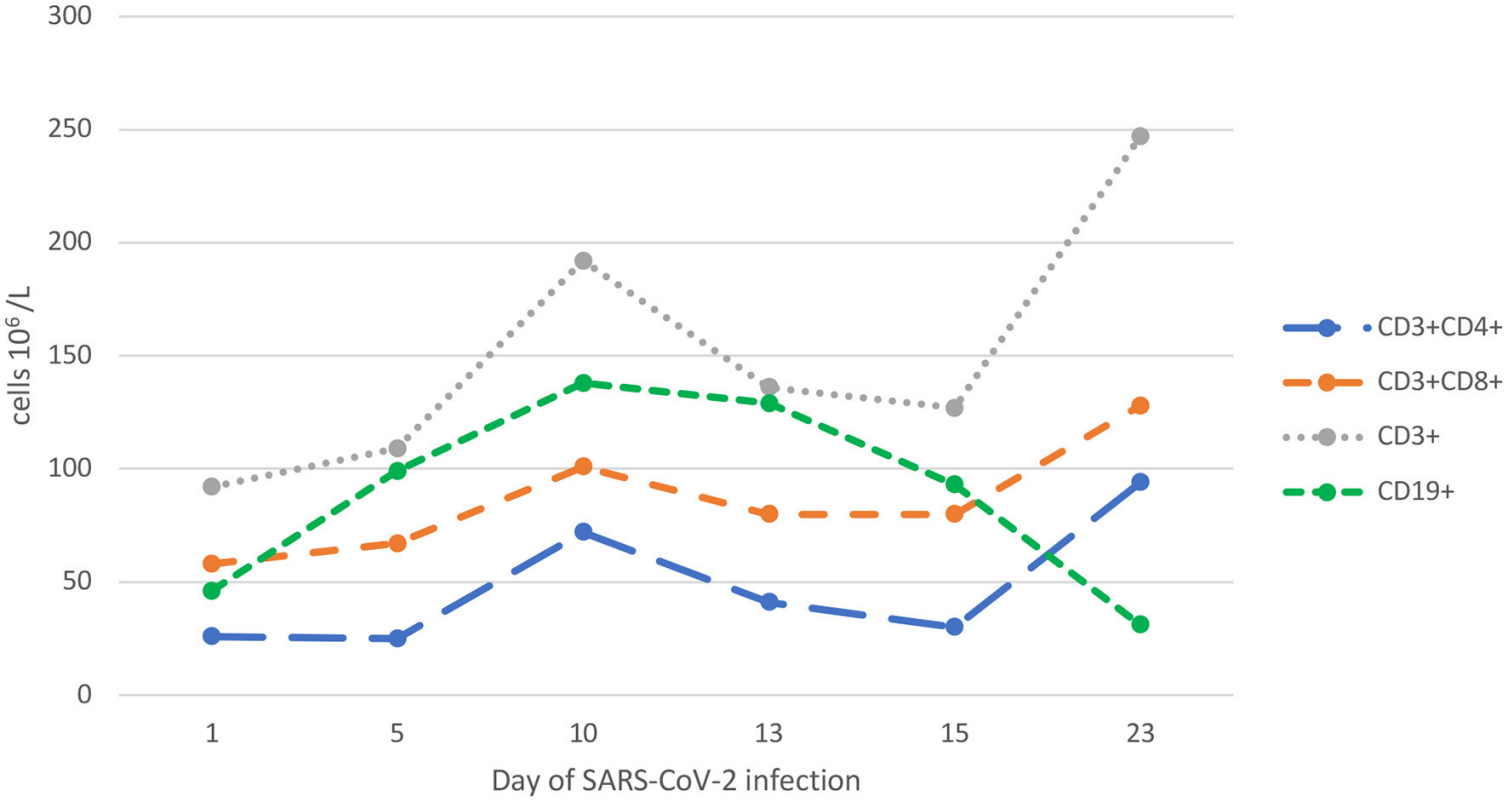
Dynamic lymphocyte parameters during SARS-CoV-2 infection.

Surprisingly, SARS-CoV-2-specific neutralising antibodies could be detected within 15 days despite serious lymphopenia with B-cell dyshomeostasis and systemic immunosuppression with prednisone. The increasing level of neutralising antibodies correlated with clearance of the SARS-CoV-2 load within 23 days and the expansion of T-cell subpopulations, as shown in Figures 2, 3. The patient's symptoms resolved completely.

At day +274 (23 days after diagnosis of SARS/CoV-2 infection), coagulation parameters and triglycerides were in the normal range. White blood count, platelets and lymphocyte subsets increased with a tendency toward normal ranges in all other laboratory tests as shown in Table 1. Of note, an autoantibody expression test was negative and there were decreased circulating CD21^low^ B cells. In parallel with these signs of immunological normalisation, the patient cleared EBV DNAemia (Figure 2). Thereafter, we continued ECP and tapered prednisone slowly. Three months later (day +365), chest computed tomography evidenced complete resolution of the SARS-CoV-2-associated pulmonary changes.

At day +296, 44 days after diagnosis of SARS-Cov-2 infection, complete response of active cGVHD manifestations was observed, with mild, irreversibly reduced range of motion and mild dyspigmentation of the skin of the forearms.

## Discussion

This case report describes a moderate course of SARS-CoV-2 infection in a patient <9 months after HSCT who had active, severe cGvHD and was undergoing systemic immunosuppressive treatment. Notwithstanding serious immune dyshomeostasis with autoantibody expression, the patient developed a sufficient humoral immune response against SARS-CoV-2 to clear this infection. Furthermore, concomitant EBV reactivation resolved without development of disease despite profound T-cell lymphopenia. Despite our concerns that infection would exacerbate cGvHD, parameters of immune dysregulation with autoantibody expression normalised and were followed by resolution of cGvHD thereafter.

In a retrospective, multicentre study, Passamonti et al. reported a worse outcome of SARS-CoV-2 infection in 536 adult HSCT survivors in comparison to the general population in Italy (17). Summarising risk factor analyses from Italian and Spanish centres, an interval from HSCT beyond 12 months, progressive primary disease status, increasing age, and arterial hypertension are risk factors for poor outcome of SARS-CoV-2 infection (17, 18). Additionally, Shah et al. described an association between the number of comorbidities, chest infiltrates and neutropenia with an unfavourable outcome of SARS-CoV-2 infection in 77 adult patients after cellular therapy (19). An observational Centre for International Blood and Marrow Transplant Research (CIBMTR) study included 318 HSCT recipients with a SARS-CoV-2 diagnosis and confirmed the association of the time interval from HSCT <12 months and age with higher risk of mortality. Of note, a mild SARS-CoV-2 disease course was observed in 49% of patients (20). Varma et al. found that concomitant steroid treatment was an additional risk factor for poor outcome of SARS-CoV-2 infection in adult HSCT patients (21). Recently, Sahu et al. comprehensively reviewed the challenges, risk factors and outcomes of SARS-CoV-2 infection in HSCT patients: active, prolonged immunosuppression and GvHD put patients at higher risk of SARS-CoV-2 infection (12).

Our case illustrates an attenuated SARS-CoV-2 infection in an immunocompromised patient for whom several risk factors for unfavourable outcome were identified such as malignant primary disease (ALL) with pre-HSCT treatment, myeloablative conditioning, recent HSCT (9 months), neutropenia, and active cGvHD with concomitant steroid treatment.

Siddiqi and Mehra hypothesised that there are two pathologic processes in patients with SARS-CoV-2 infection: the first is triggered by the virus itself and the second by the host response (22). They proposed a triphasic clinical staging system: stage I (early infection), stage II (pulmonary phase) and stage III (hyperinflammatory phase) with associated phase-specific signs, symptoms and possible therapeutic targets. Stage I seems to be similar in immunosuppressed and non-immunosuppressed patients, while stages II and III seem to be milder and less frequent in immunosuppressed individuals.

In non-immunocompromised patients, an observed significant decrease in total number of lymphocytes has suggested that lymphocytes, particularly T lymphocytes, are likely targets of SARS-CoV-2 (23). In line with this, de Candia et al. reported that the dysregulation of the innate and adaptive immune system with cytokine storm and deterioration of T-cell response is an essential factor for morbidity in SARS-CoV-2 infection (24). Summarising published evidence Sahu et al. confirmed thrombocytopenia, elevated D-dimers and lymphopenia to be associated with poor prognosis of SARS-CoV-2 infection in immunocompetent patients (12). However, the extrapolation of findings from non-HSCT to HSCT patients seems difficult.

In contrast, studies in paediatric immunocompromised patients showed that T-cell lymphopenia was associated with fewer severe morbidities such as acute respiratory distress syndrome and hyperinflammation (20). We speculate that severe lymphopenia and cGvHD treatment with 0.8 mg/kg/day prednisone and ECP had a protective effect with an attenuated inflammatory response in our patient. Although laboratory assessments revealed signs of MAS, the latter was not represented in clinical symptoms of a cytokine storm. For this reason and because no anti-SARS-CoV-2 mAbs were available at that time, no further therapy was implemented. ECP had to be postponed for 4 weeks due to logistical reasons.

The role of B-cell dyshomeostasis in cGvHD has been reported by several groups, primarily in adult patients (7, 25). Recently, our group and Schultz et al. were able to confirm similar findings in prospective studies of paediatric cGVHD patients (9, 10). Furthermore, Kuzmina et al. described autoantibody expression in patients with cGVHD as a biomarker for autoimmunity and immunodeficiency (8).

Our patient suffered from severe multiorgan cGvHD with signs of B-cell perturbation including autoantibody expression and elevated CD21^low^ B cells. In cGVHD, a reduction in antibody response to neoantigens and a lack of class switch from IgM to IgG is well-known (26). Surprisingly, our patient was able to produce SARS-CoV-2 antibodies. Shah et al. reported that several HSCT patients who lacked circulating B cells were able to develop SARS-CoV-2 antibodies, suggesting antibody production from non-circulating lymph node or tissue-resident cells (19).

In SARS-CoV-2, various autoantibodies have been described and associated with severe disease and the development of autoimmune pathologies (27–29). These findings are consistent with the claim that SARS-CoV-2 has the ability to hyper-stimulate the immune system (30). Unexpectedly, our patient cleared all autoantibodies after SARS-CoV-2 infection, with improvement of immune parameters of the B- and T-cell compartment. We can only speculate that SARS-CoV-2 stimulated but not hyper-stimulated an otherwise aberrant immune system.

The interplay between infection and cGvHD may be mutual: antigenaemia and an inflammatory environment stimulate the development or exacerbation of cGvHD, while immunodeficiency related to cGVHD itself and its treatment favour reactivation and infection (31, 32). We assume that the immunomodulatory effect of ECP as treatment pre and post SARS-CoV-2 infection (together with prednisone) may have allowed the development of immune tolerance with sustained control of severe cGvHD without further risk of infection or ALL relapse (33). Foss et al. reported a case of attenuated SARS-CoV-2 infection in a patient with severe cGvHD treated with ruxolitinib and ECP (34). The authors suggested that ECP or ruxolitinib may have played a role in reducing the inflammatory response. Of note, although the various immunomodulatory mechanisms of ECP for the prophylaxis/treatment of GvHD have been well-published (35), these benefits do not necessarily translate to SARS-CoV-2 infection.

Some authors have observed herpes virus reactivation during SARS-CoV-2 infection in non-compromised patients as an expression of immunodeficiency. Chen et al. reported a co-reactivation of EBV in 50% of patients with SARS-CoV-2 infection (36). Furthermore, Lehner et al. observed EBV DNAemia in 78% of patients with SARS-CoV-2 infection admitted to the intensive care unit (37). An association between EBV reactivation and the severity of CD3^+^CD8^+^ T-cell lymphopenia was reported by Liu et al. (38). In our patient, we interpreted the short duration of EBV viraemia without any clinical signs of EBV disease as an indicator of the patient's severe immunodeficiency caused by SARS-CoV-2 infection on top of cGvHD and its treatment. Of note, both donor and recipient were EBV antibody positive before HSCT. Our patient mutually cleared SARS-CoV-2 and the EBV infection. Furthermore, he never experienced SARS-CoV-2 reactivation despite ongoing immunosuppression.

In conclusion, this case suggests that lymphopenia and systemic immunosuppression for active cGvHD at the onset of the SARS-CoV-2 infection 9 months after HSCT might not be a risk factor for an unfavourable outcome. Furthermore, it emphasises the need for close monitoring of additional viral complications during SARS-Cov-2 infection. Our report demonstrates that, despite serious immune dyshomeostasis with autoantibody expression, the patient mounted a sufficient humoral immune response to clear the infection. Moreover, we observed resolution of cGvHD thereafter with ECP and prednisone treatment, although we cannot exclude other contributing factors.

## Data Availability Statement

The original contributions presented in the study are included in the article/supplementary material, further inquiries can be directed to the corresponding author/s.

## Ethics Statement

Written informed consent was obtained from the individual(s) for the publication of any potentially identifiable images or data included in this article.

## Author Contributions

NZ and AL wrote the case report. IH-P supported technical aspects of the report. IH-P, MI, and OH critically appraised the first and final drafts. All authors contributed to the article and approved the submitted version.

## Conflict of Interest

The authors declare that the research was conducted in the absence of any commercial or financial relationships that could be construed as a potential conflict of interest.

## Publisher's Note

All claims expressed in this article are solely those of the authors and do not necessarily represent those of their affiliated organizations, or those of the publisher, the editors and the reviewers. Any product that may be evaluated in this article, or claim that may be made by its manufacturer, is not guaranteed or endorsed by the publisher.
